# Calculation of partial isotope incorporation into peptides measured by mass spectrometry

**DOI:** 10.1186/1756-0500-3-178

**Published:** 2010-06-24

**Authors:** Ingo Fetzer, Nico Jehmlich, Carsten Vogt, Hans-Hermann Richnow, Jana Seifert, Hauke Harms, Martin von Bergen, Frank Schmidt

**Affiliations:** 1 Helmholtz Centre for Environmental Research - UFZ, Department of Environmental Microbiology, Permoserstrasse 15, D-04318 Leipzig, Germany; 2 Helmholtz Centre for Environmental Research - UFZ, Department of Proteomics, Permoserstrasse 15, D-04318 Leipzig, Germany; 3 Helmholtz Centre for Environmental Research - UFZ, Department of Isotope Biogeochemistry, Permoserstrasse 15, D-04318 Leipzig, Germany; 4 Interfaculty Institute for Genetics and Functional Genomics, University of Greifswald, Friedrich-Ludwig-Jahn-Strasse 15a, D-17487 Greifswald, Germany

## Abstract

**Background:**

Stable isotope probing (SIP) technique was developed to link function, structure and activity of microbial cultures metabolizing carbon and nitrogen containing substrates to synthesize their biomass. Currently, available methods are restricted solely to the estimation of fully saturated heavy stable isotope incorporation and convenient methods with sufficient accuracy are still missing. However in order to track carbon fluxes in microbial communities new methods are required that allow the calculation of partial incorporation into biomolecules.

**Results:**

In this study, we use the characteristics of the so-called 'half decimal place rule' (HDPR) in order to accurately calculate the partial^13^C incorporation in peptides from enzymatic digested proteins. Due to the clade-crossing universality of proteins within bacteria, any available high-resolution mass spectrometry generated dataset consisting of tryptically-digested peptides can be used as reference.

We used a freely available peptide mass dataset from *Mycobacterium tuberculosis *consisting of 315,579 entries. From this the error of estimated versus known heavy stable isotope incorporation from an increasing number of randomly drawn peptide sub-samples (100 times each; no repetition) was calculated. To acquire an estimated incorporation error of less than 5 atom %, about 100 peptide masses were needed. Finally, for testing the general applicability of our method, peptide masses of tryptically digested proteins from *Pseudomonas putida *ML2 grown on labeled substrate of various known concentrations were used and^13^C isotopic incorporation was successfully predicted. An easy-to-use script [1] was further developed to guide users through the calculation procedure for their own data series.

**Conclusion:**

Our method is valuable for estimating^13^C incorporation into peptides/proteins accurately and with high sensitivity. Generally, our method holds promise for wider applications in qualitative and especially quantitative proteomics.

## Background

An important aspect of microbial ecology is to link specific microorganisms to microbially-driven processes in the natural environment [2]. Indeed, these questions can be addressed with more accuracy by using isotopically labeled substrates to follow metabolic carbon flux within microbial cultures. Subsequently, the incorporation of the label into the biomass may be followed by analysis of either fatty acids [3], deoxyribonucleic acid (DNA) [4] or ribonucleic acid (RNA) [5]. Unfortunately, those techniques are limited in their ability to resolve low levels of isotopic labeling and their absolute incorporation. Essentially, the information about low level labeling as well as the assignment of distinct incorporation levels to various species is needed in order to understand the metabolic interdependencies within complex microbial communities.

**Figure 1:**
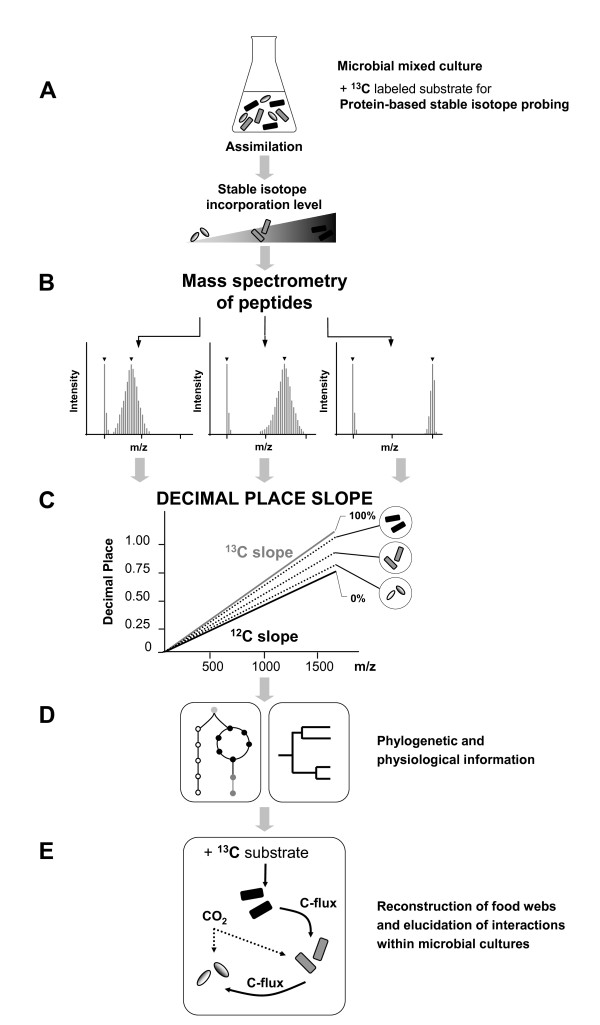
**Schematic overview about the workflow of analysis**. (**A**) Assimilation of heavy stable isotopes into the biomass of various species depends on the turnover and the interaction activity of the species. The incorporation of stable^13^C isotopes from a substrate can be used to pinpoint the metabolically active species within a consortium. The different incorporations are indicated by various amounts of label (gray-color scale). (**B**) After cell harvesting and protein extraction, samples were tryptically digested and analyzed by MS. The isotopologues shifted to a higher mass range due to their incorporation of heavy labeled carbon into the proteins. A higher level of incorporation indicates a faster growth rate and/or a primary role in the degradation of the labeled substrate within the food web. (**C**) The incorporation of heavy stable isotopes into peptides/proteins can be estimated using the HDPR. (**D**) Peptides can be used to obtain phylogenetic information (in case of unique peptides), for structural analysis, and for physiological information about the actual state of the microbial cells. (**E**) Based on this information, C-fluxes and food web structures can be elucidated and may further help to reconstruct the interaction of microbial communities.

To overcome the drawbacks of other calculation methods, we recently developed a new protein-based stable isotope probing (Protein-SIP) technique [6]. A brief overview of the workflow is displayed in **Figure **1. In short, the incorporation of stable e.g.^13^C and/or^15^N isotopes from a substrate was used to pinpoint the metabolically active species within a consortium. Different species incorporate different degrees of the stable isotopes into their biomass according to their physiological ability and their activity (**Figure **1A), which can be detected on peptide/protein level [7]. After cell harvesting and protein extraction, samples were analyzed by various proteomic and mass-spectrometry techniques. Due to the isotopic abundance of^13^C and/or^15^N, several isotopic envelopes can be detected by high-resolution mass spectrometry (MS) [8,9]. As expected, the isotopologues shifted to a higher mass range due to the incorporation of heavy labeled carbon into the proteins, as shown in the MS-spectrum in **figure **1B. The^12^C-containing peptides are shown at the left hand side of each spectrum. A higher level of incorporation, as shown in the right spectra of part B, indicates a faster growth rate and/or an increased substrate usage of the carbon source of species. In order to assign these activities to different species, two steps have to be applied (i) the light peptides have to be identified by Tandem-MS (mass spectroscopy) and further assigned to the bacterial taxon and (ii) the heavy stable isotope incorporation has to be determined.

One method of calculation is to compare^12^C and^13^C isotopologue distributions in one MS-spectrum and estimate^13^C incorporation by calculating the mass difference between the monoisotopic^12^C mass peak and the highest mass peak of the^13^C isotopologue. This procedure depends on a proper pre-fractionation, since the corresponding mass peaks can be rather difficult to identify. Alternative calculation methods are necessary to find the matching pairs at low incorporation levels [10].

Therefore, an improved method based on the comparison of theoretical and experimentally determined isotopic envelopes of peptides with known sequences was developed [11,12]. However, both methods require *a priori *peptide identification to accurate determine the dynamic labeling of heavy stable isotopes. In order to reliable quantify partial^13^C incorporation into peptides, we developed a method based on the "half decimal place rule" (HDPR) (**Figure **1C). Information about the dynamic incorporation levels in proteins from different microbial species can be used to elucidate the structure and function of the microbial community (**Figure **1D). With this information, the carbon fluxes throughout the community may be followed and enables to deduce species interactions and activities (**Figure **1E) [13,14].

In this study, we describe a new and easy-to-apply algorithm for the determination of absolute heavy stable isotope incorporation (^13^C) into peptides/proteins by taking advantage of high-resolution MS data [15,16], and the characteristic pattern arising from the decimal residuals of incorporated heavy stable carbon isotopes [17]. Annotated sequence information for measured peptides is no longer necessary for the calculation of^13^C incorporation that is an important consideration when analyzing uncultivable or incompletely sequenced bacteria. Moreover, we provide an easy-to-apply script set written in the statistical programming language R [18], enabling scientific researchers to calculate^13^C incorporation into peptides/proteins.

### Theoretical background

In the past, mass spectrometers suffered from their low resolution and accuracy, restricting the possibility to predict chemical element contents based on the mass signals. Higher accuracy allows to create exact fitting curves describing the behavior of a mass signal as a function of both the mass and the e.g. sulfur content [19]. With up-coming of high resolution MS devices, a linear relationship between tryptic peptide masses and the decimal residuals (=digits behind the mass' decimal point) has been firstly observed by Mann [20] (for additional references see [19,21]). Closer investigation of this mass mapping phenomenon by Schmidt *et al*. [17], led to the definition of the linear relationship of tryptic peptides and the corresponding decimal places, the so-called "half decimal place rule" (HDRP).

This rule declares that the decimal place of a tryptic peptide is near the half of the first digit for tryptic peptides in the range of 500-1,000 Dalton (Da), near the half of the first two digits for the range of 1,000-1,999 Da and again near the half of the first digit for masses from 2,000-3,000 Da. The rule was found to be helpful for e.g. the detection of non-peptide contaminants in mass spectrometric measurements and the recalibration of peptide masses [17]. Since only the digits behind the decimal point are necessary as information in order to identify some artifacts of tryptic peptides.

During the metabolic incorporation of^13^C amino acid precursors into proteins,^13^C atoms are gradually replaced^12^C atoms. This elemental substitution increases each peptide mass by exactly 1.003355 Da (= the difference between^12^C and^13^C atomic mass) per substituted carbon atom. In terms of decimal places for peptides, each incorporated heavy carbon atom result in a mass shift of exactly 0.003355 Da. Applying the HDRP to partly or fully labeled peptides, the linear relationship between peptide masses and their decimal places will result in an increase of the corresponding slope. The steepness of the slope increases in proportion with the amount of incorporated heavy stable isotopes. This effect can be used for the exact estimation of the amount of incorporation for any given set of measured peptide masses. The detection of these small mass shifts, however, requires highly accurate measurements. This is now possible with modern mass spectrometers such as a fourier transformation ion cyclotron resonance (FT-ICR), linear ion trap with an Orbitrap (LTQ-Orbitrap) or quadruple time-of-flight (Q-TOF) instrument.

## Methods

### Calculation of isotopic composition of peptides

#### Processing of reference dataset

Since proteogenic amino acids are universal within all bacterial species, any high-resolution dataset can be used for referencing the theoretical decimal place calculation (as reference data and for calibration). An existing tryptic peptide dataset of *Mycobacterium tuberculosis *H37Rv was used [22] that originally contained ~4,000 encoding genes of which 3,924 were identified as proteins [23]. The complete FASTA protein dataset is freely available from the Sanger Institute [24]. After a tryptic *in-silico *digest by MS-Digest [25], peptides between the mass range *m/z *300-6,000 were considered for further analysis, resulting in 315,579 peptide fragment sequences. Further, we restricted the dataset to include only those tryptic peptides with a ChemScore ≥ 10 (ChemScore = sum of total free protein binding energy (for further details see [26-28]) and without missing cleavage sites or modifications. After this screening, the dataset was reduced to 90,637 remaining peptide sequences containing lengths between 2 and 40 amino acids. The monoisotopic mass of each sequence was calculated with given atomic masses of^12^C = 12.000000 Da,^14^N = 14.003074 Da,^16^O = 15.994915 Da,^1^H = 1.007825 Da,^32^S = 31.972071 Da to obtain the masses of 'light' (0 atom %^13^C incorporation) amino acids. Subsequently, by counting the numbers of carbons for each sequence and replacing the^12^C mass for the heavier^13^C (13.003355 Da), a dataset for the theoretical 100 atom %^13^C incorporation was obtained. The advantage of calculating the weight by counting C atom numbers is that the same procedure can be used to easily calculate a wide range of different^13^C incorporation levels.

#### Data classification and modification

**Figure 2:**
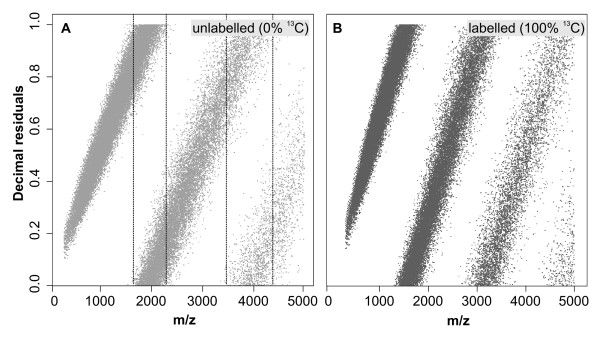
**A scatter plot of masses from 90,637 peptides (*m/z*) and decimal residuals (digits behind the decimal point) of *Mycobacterium tuberculosis *within a mass range of *m/z *0-5,000**. (**A**) Unlabeled sequences (0 atomic %^13^C incorporation), (**B**) fully labeled (100 atomic %^13^C incorporation). Lines depict the overlap of scatter groups.

Direct plotting of peptide masses against their decimal residuals would result in regular, diagonal linear shaped patterns (**Figure **2). With increasing peptide masses, also their decimal residuals increase until they approach values close to 1. With further increasing, the decimal residuals start with values of 0 again. In order to obtain a continuous plot, the points of the second band had to be added on top of the first, and those of the third band on top of the second etc. by subsequently adding 0, 1, 2, etc Dalton to the residuals of each band.

**Figure 3:**
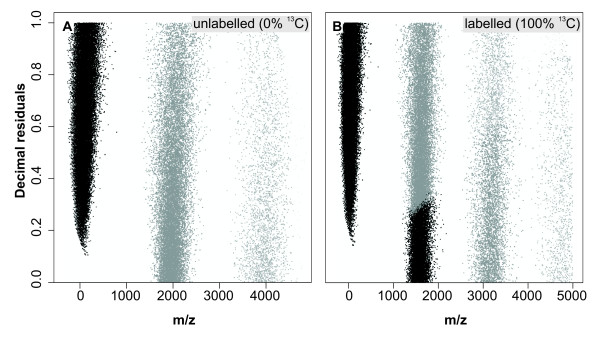
**A scatter plot of temporally transposed masses from 90,637 peptides (*m/z*) and decimal residuals of a *Mycobacterium tuberculosis *dataset (details see text)**). Grey dots indicate cluster affiliation after classification by k-means clustering for (**A**) Unlabeled sequences (0 atomic %^13^C incorporation), (**B**) fully labeled (100 atomic %^13^C incorporation).

**Figure 4:**
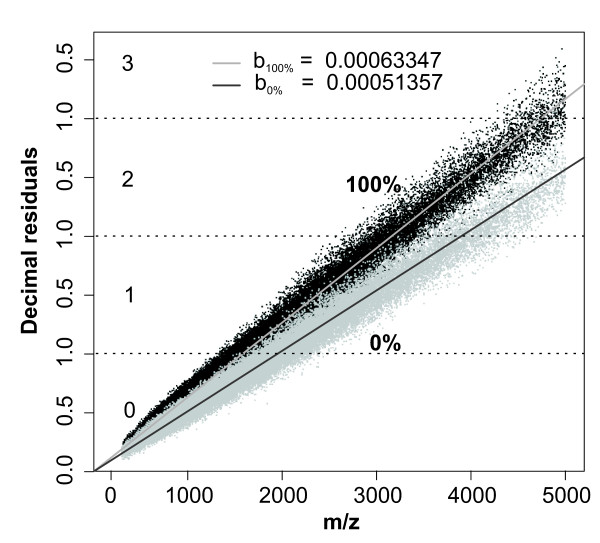
**A scatter plot of 90,637 peptide masses (*m/z*) and their decimal residuals of a *Mycobacterium tuberculosis *dataset after classification and data modification for unlabeled (0 atom %^13^C incorporation; light grey) and fully labeled (100 atom %^13^C incorporation; dark grey) peptide masses resulted**. The two lines represent the slopes for the calculation of^13^C incorporation. The values of the two scatters are for the unlabeled b_0% _= 5.1357e-4 and b_100% _= 6.3347e-4 for fully labeled dataset. Numbers in each segment represent Daltons added to decimal residuals to achieve straight plot from figure 3 (for details see text).

However, due to slight deviations from linearity caused by the amino acid residues, an overlap of the bands occurs (indicated by vertical lines in **Figure **2A), disabling a direct distinct and straightforward separation. Moreover, increasing substitution of peptides with heavy stable^13^C isotopes does not only result in an alteration of the slope becoming steeper, but also produces a continuous and gradual shift of the band along the *m/z *axis. Therefore, we developed a semi-automatic and more flexible approach. Each point cloud was separated by a standard classification method using k-means clustering. Prior to clustering, we transformed the original mass values in relation to their decimal residual values by the following equation,(1)

where P_M Trans _= transposed peptide masses, P_M _= peptide masses, and D_R _= decimal residuals producing a plot as given in **Figure **3. The value of 1,800 within the formula (1) was iteratively estimated. Gradually increase of this value made the bands steeper until reaching a maximal vertical position at a value of about 1,800. A value greater than 1,800 made the bands tilt towards the opposite direction. The following classification was conducted using the Hartigan and Wong algorithm for k-means clustering [29] with three (0; 2,000; 4,000 Da) and four (0; 1,600; 3,200; 4,800 Da) pre-set clustering centers for the 'light' (no^13^C incorporation) and 'heavy' (complete^13^C substitution) dataset. For the clustering, pre-set center mass values do not have to be overly accurate since the precise numbers are automatically determined during the clustering procedure from actual group means [30]. Finally, the original peptide masses were taken and ranked according to cluster affiliation of the corresponding transformed mass values. In a next step, 0 Da was added to the peptide mass values belonging to the first cluster, 1 Da was added to the values of the second cluster and so on. Plotting these new mass values versus their decimal residuals resulted in the final straight plot (**Figure **4).

#### Data plotting and linear slope estimation

Following this principle for peptide masses relating to 0 atom % and 100 atom %^13^C incorporation, two uniformly increasing point clouds with dissimilar slopes were produced (**Figure **4). For both datasets, a fit on a linear model given by *D*_*R *_= b*P_*M *_with b equal to the slope value and an intercept of zero was carried out. The slope value b was estimated by adjusting the slope and minimizing the error sum of squares (SSE) of the decimal residual deviations:(2)

with slope b calculated as:(3)

with D_R _= decimal residual, P_M _= peptide mass, and n = number of peptide masses [31]. Slopes for 0 atom % and 100 atom %^13^C incorporation were estimated with an accuracy of 6 decimal digits and served as references for partial^13^C incorporation calculation.

All methods described above are summarized as easy-to-follow R-scripts which can be run under the statistical platform R [18]. 'Script 1' enables calculation of own reference dataset for 0% atom and 100% atom^13^C from any other protein mass dataset while reference slope calculation of can be done with help of 'Script 2'. Both scripts can be downloaded under [1].

### Calculation of^13^C incorporation from practical mass spectrometric measurements

In order to estimate^13^C incorporation from experimentally derived datasets, processing occurred as described above. However, k-means clustering with very small datasets sometimes failed, because the algorithm required that at least one data point in each cluster to be present. In order to circumvent this difficulty, a set of dummy data containing one data point with values at the exact centers were added. Prior to^13^C incorporation calculation, those dummy points are removed again and, thus, have no influence on the following calculation.

In a second step, a linear fitting was conducted. While the standard linear fit by minimizing SSE is sufficient for large datasets, it is not feasible for smaller datasets like many user data consisting of relatively few peptide masses. Moreover, these data are often irregularly distributed and can contain outliers with extreme values characterized by strong deviations from linearity. In such cases, standard linear fitting is neither appropriate nor robust. Moreover, commonly applied SSE-based methods rely on the assumption that data residuals are normally distributed that, within user data is rarely found in practice. With datasets containing high variability and/or outliers, classical linear approximation methods often perform poorly [32,33]. Therefore, we applied the more accurate and robust linear fitting algorithm (*rlm*) provided by the MASS package [30] in R that are better suited for small and highly variable datasets.

The rlm fitting approximation is using an iterative process examining which points cause strong shifts in the slope, and inversely re-weighting the data points during the least squares fitting process (IWLS-method [32,34]). Using likelihood estimators, statistically robust methods minimize small deviations from model assumptions [30,34].

Using reference slope values estimated (**Figure **4) the relative^13^C incorporation of practical datasets is finally calculated by the relative fraction of the practical dataset(4)

with^13^C_User _% = relative isotope incorporation values from user data, b_user _= slope of regression estimated for practical data, b_12C _and b_13C _=^12^C and^13^C regression slope value for 0 atom %^13^C and 100 atom %^13^C incorporation, respectively.

We developed an easy-to-use R script for the estimation of^13^C atom % incorporation for user datasets which can be downloaded under [1] as 'script 3'.

User data have to be provided as an ASCII .txt file. The file should have the values of the peptide masses containing one peptide mass (z = 1 after deconvolution) per line with no header and/or row names. Measurements can be provided with comma or point decimal markers.

**Figure 5:**
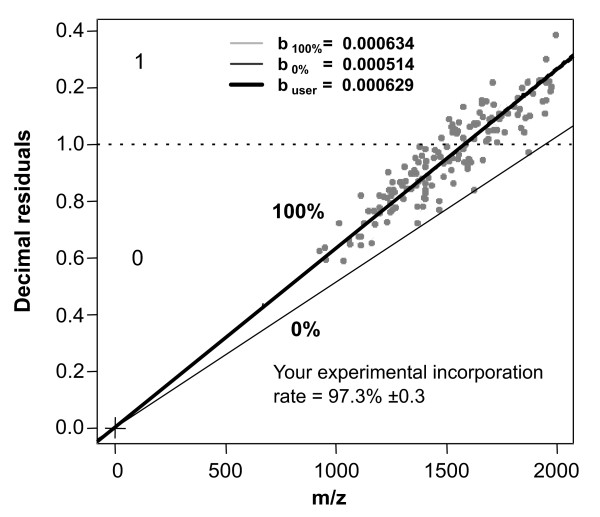
**Typical plot resulting from user experimental measurements (dark circles), reference slope lines for unlabeled (0 atom %^13^C incorporation; dark grey line) and fully labeled (100 atom %^13^C incorporation; light grey line) and the slope calculated from user data measurements (thick black line)**. The^13^C incorporation for user data is directly calculated and displayed in the plot. This example consists of 150 practical peptides from *Pseudomonas putida *grown on fully labeled benzene.

During the run of the script, a window opens and the user has to indicate where the ASCII.txt file is located. The file should have the values of the peptide masses containing one peptide mass (z = 1 after deconvolution) per line with no header and/or row names. After selecting the file, the calculation starts immediately and results in a scatter-plot with lines displaying the 0 atom % and 100 atom % incorporation reference lines, the user's peptide masses, the fitting line of the data with the standard error as quality proxy, and the estimated incorporation including the standard error estimation (**Figure **5). In a loop process, the estimation of multiple measurement campaigns can be carried out.

### Accuracy of the method

**Figure 6:**
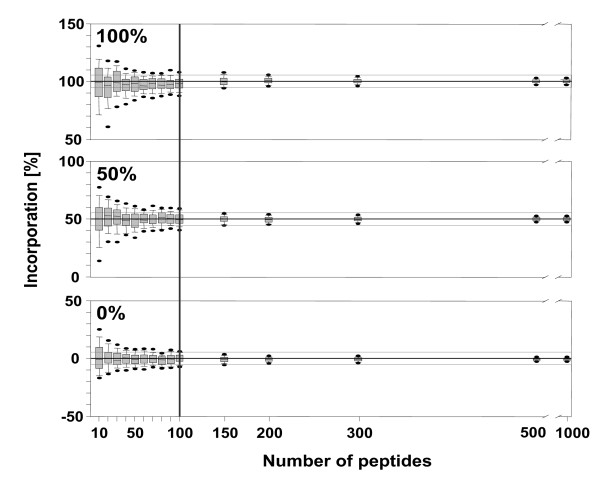
**Box-and-whisker plot for approximate accuracy of^13^C incorporation for groups of 10, 20, 30, 40, 50, 60, 70, 80, 90, 100, 200, 300, 500, and 1000 peptides from 100 random draws (no repetition) for *Mycobacterium tuberculosis *peptide dataset of 0, 50 and 100 atom %^13^C incorporation**. Boxes represent lower and upper quartile, whiskers the upper and lower (95%) confidence interval and thick lines in boxes the median within the sub-sample groups. The two thin black lines depict 5% incorporation limits and the thick black line depicts the number of samples necessary to cross the 5% incorporation limit.

In order to determine the minimal number of needed peptide masses with an estimated incorporation accuracy of less than 5%, we additionally calculated an *in silico *peptide dataset with 50 atom % isotope incorporation of *M. tuberculosis*. We sub-sampled then the two already existing 0 and 100 atom, and the newly created 50%^13^C datasets by randomly drawing groups of 10, 20, 30, 40, 50, 60, 70, 80, 90, 100, 200, 300, 500, and 1,000 theoretical peptides across all peptide lengths with no repetition for 100 replications. For each within-group sub-sample, we calculated the median and the standard error of each subgroup. A resulting box-and-whisker plot for each drawing shows the accuracy of the incorporation as a function of number of peptide masses (**Figure **6).

### General applicability of the method

The general applicability of our method was tested using a dataset from *Pseudomonas putida *ML2 growing on fully labeled^13^C-benzene (0.6 mM) until the stationary growth phase. The detailed experimental conditions are explained by Jehmlich *et al*., [6]. After protein extraction, peptides were analyzed by nano-LC-LTQ Orbitrap-MS. From these dataset, information form 150 tryptically-digested peptide masses were taken and the heavy stable isotopes incorporation was estimated.

## Results and Discussion

### Data processing

For the calibration of the unlabeled and completely labeled incorporation, a dataset of *M. tuberculosis *containing atomic masses of > 90,000 peptide fragments were used. Plotting unlabeled peptide masses directly against their decimal residuals. As a result, up to *m/z *values of ~2,000 a first point cloud increased linearly with corresponding decimal residuals approaching values close to 1 (**Figure **2A). When the decimal residuals reaching 1, the next residual digits start to fill up and thus start at 0 again. Therefore, increases across values of 1 leads to the development of the next point cloud with values close to 0. For *m/z *ranges between 300 and 5,000, new point clouds begin. Especially, for fully labeled peptide masses, these breaks occur at slightly lower values of ~1,800 Da, ~3,200 Da, and ~4,800 Da, since overall slopes of scatter plots here become slightly steeper (**Figure **2B). However, variations in the molecular composition of each theoretical peptide mass impeded a simple separation into groups in order to create a continuous linear plot. Therefore, transient transformation using formula (**1**) was required. During the transformation process, peptide masses with small residual values (i.e. those close to 0) are comparatively altered very little, while those with big values (close to 1) experience strong shifts towards the ordinate and thus result in the expected increase of slope steepness for each group (**Figures **3A and 3B). Subsequent to transformation with formula (**1**), allows straightforward separation of the point clouds for both the unlabeled and the fully labeled dataset, and the clustering algorithm was able to separate all data points into distinct classes. Misgrouping occasionally occurred with higher peptide masses, where points become more dispersed within the group. However, misgrouping occurred only in less than 0.1% of all data points.

After separation into groups, and the addition of 0, 1, 2 and 3 Da according to the group affiliation of each data point, two separated linear point clusters with distinctive slopes were obtained (**Figure **4). Two characteristics can be observed: (**i**) the two point clouds display heteroscedastic characteristics. The initially compact plot becomes more dispersed at higher peptide masses, with higher inclination from the ideal linearity. However, peptide masses have increased variance; (**ii**) since the used database also contains short-chained amino acid sequences, the point clouds also become less dense with increasing peptide masses.

### Linear slopes and estimation of relative isotope incorporation

The accurate slope value for the unlabeled dataset was estimated as b_0% _= 5.1357e^-4 ^and for the fully labeled dataset b_100% _= 6.3347e^-4^. These two values were used in further calculations as our standards for the 0 atomic % and 100 atomic % incorporation.

By applying formula (**4**), we were able to calcuate incomplete heavy stable isotope incorporation into peptides of user data. However, standard linear fitting depending on minimizing SSE turned out to be strongly influenced by outliers. Strong deviation of the expected slope caused by outliers occurred most frequently in scenarios with inhomogeneous distribution of the measurements along the *m/z *range. In the presence of outliers, especially with high values, traditional methods are inefficient and biased because the least squares predictions are often heavily dragged towards these outliers. While every added point in the traditional SSE method has a direct influence on the slope, SSE has a breakdown point value of 0 and signifying that every added point has the same influences on the slope value. Our recommended robust linear model algorithm has a breakdown point value of 0.5, meaning that at least 50% of the data need to be altered to cause the slope estimation to change. Thus, when peptide masses with high mass strongly deviated from the linear reference slope, the final slope completely affected by these values. Using the *robust linear fitting *(rlm) algorithm, the slope for the calculation can only altered if more than 50% of all used data points varied. Therefore, the rlm algorithm is more robust and stable if outliers are used in the dataset.

### Accuracy of method

The accuracy of incorporation is strongly depends on the considered number of peptide masses. As expected, the accuracy was very poor with small datasets (when using only 10-30 peptide masses). The prediction accuracy was only < 25% at known 0, 50, and 100 atom %^13^C incorporation (**Figure **6). However, after including more than 50 peptides, the prediction asymptotically approached less than 10% precision. After incorporation of about 100 peptide masses, the accuracy became better than 5%. However, the inclusion of more peptide masses (> 200) did not improve the accuracy further.

### General applicability of the method

The method can be applied to a broad range of bacterial taxa. Data from other bacteria than the *M. tuberculosis *reference strain proved also to be very successful. In order to validate the method, we used peptides of the bacterium *P. putida *ML2 grown on fully labeled substrate in a batch culture. Obtained mass spectra were analyzed by a LTQ-Orbitrap and the incorporation of^13^C into the peptides was calculated applying our method. After data points fitting a graph with a slope of 6.2910e^-4 ^was generated that corresponds to 97.3% ± 0.3%^13^C incorporation. This value of^13^C incorporation is quite comparable with other MS-based methods where the labeling efficiency was estimated as 98.6% ± 0.2% for^13^C labeled peptides [35]. However, in comparison of our algorithm with other MS-based methods, generally for the latter a much higher effort for estimating incorporation values is needed.

## Conclusion

We demonstrated that our method can be used to estimate the^13^C incorporation into peptides/proteins accurately and sensitively. The method requires about 100 peptide masses in order to achieve an accuracy of less than 5%.

Generally, our method holds promise for wider applications in qualitative and quantitative proteomics. Comprehensive proteomic analyses of mixed communities would enable microbial ecologist to link community composition, physiology, function, ecology, interaction, and evolutionary processes [36]. Metaproteome analyses were performed by either gel-based [37] or by LC-MS based approaches [38] and would go one-step further with the combination of metabolic labeling and proteome analysis (Protein-SIP) by obtaining the metabolic activity of various species in microbial communities.

## Competing interests

The authors declare that they have no competing interests.

## Authors' contributions

IF participated in the conceptual design of study, conducted the computational implementation of the algorithm, conducted all calculations and statistical analyses. He also implemented the method into 'R' and wrote the scripts for user application of the method. Together with NJ, he drafted the manuscript. NJ contributed to the acquisition and interpretation of protein data and isotopic measurements used for manuscript and protein data analysis. CV conducted the necessary experimental work in the lab, carried out the lab data acquisition and final interpretation of the experimental data. He also critically revised the manuscript and helped to improve it substantially. H-HR contributed in the isotopic interpretation of the data, participated in the critical revision of the manuscript and helped in the final improvement of the version for publishing. JS performed protein data analysis and did the protein data acquisition and interpretation. HH revised the MS critically and helped to restructure the MS to its final publishable version. MvB was involved in the design of the study, mainly contributed to the introductory section of the manuscript, revised the manuscript critically and participated in the final improvement of this version. FS came up with the original idea for the algorithm, participated in the design of the study and was responsible for the coordination of the study. Additionally, he substantially contributed to the writing and improvement of the manuscript.

All authors read and approved the revised final mansucript
